# Evaluation of Arterial Stiffness Parameters and the Growth Differentiation Factor-15 Level in Patients with Premature Myocardial Infarction

**DOI:** 10.3390/jpm13101489

**Published:** 2023-10-13

**Authors:** Zekeriya Dogan, Cigdem Ileri, Esin A. Kay, Murat Sunbul, Emre Y. Gurel, Beste Özben Sadıc, Nurten Sayar, Tulin Ergun, Kursat M. Tigen

**Affiliations:** 1 Department of Cardiology, Marmara University School of Medicine, Istanbul 34890, Turkey; esin.kay@gmail.com (E.A.K.); drsunbul@yahoo.com.tr (M.S.); emregurelctf@yahoo.com (E.Y.G.); besteozben@yahoo.com (B.Ö.S.); sayarnurten@gmail.com (N.S.); mktigen@yahoo.com (K.M.T.); 2 Department of Cardiology, Kosuyolu Education and Research Hospital, Istanbul 34865, Turkey; cgdmileri@gmail.com; 3 Department of Dermatology, Marmara University School of Medicine, Istanbul 34890, Turkey; tulinergun@gmail.com

**Keywords:** GDF-15, arterial stiffness, premature myocardial infarction, pulse wave velocity

## Abstract

Background: Myocardial infarction (MI) is increasing at a younger age. Growth differentiation factor-15 (GDF-15) has been implicated in several key mechanisms of atherogenesis. Arterial stiffness parameters, including pulse wave velocity (PWV) and the augmentation index (AIx), can indicate the presence or progression of atherosclerosis. The aim of this study is to evaluate the GDF-15 level and arterial stiffness parameters in patients with premature MI. Method: Thirty patients aged ≤45 years (mean age: 39 ± 5 years, 23 male) who recovered from a MI and 15 age and sex-matched subjects were consecutively included. The serum GDF-15 concentration levels and arterial stiffness parameters of the patients and controls were measured. Results: GDF-15 levels were significantly higher in patients with premature MI, while there were no significant differences in PWV and AIx between the groups. The GDF-15 level was correlated negatively with high-density lipoprotein (HDL) cholesterol and positively with uric acid levels. Both GDF-15 (*p* = 0.046, odds ratio: 1.092, 95% confidence interval: 1.003–1.196) and HDL cholesterol (*p* = 0.037, odds ratio: 0.925, 95% confidence interval: 0.859–0.995) were found as independent factors associated with premature MI. Conclusions: GDF-15 could be a risk factor for premature MI. Further studies are needed to elucidate the central role of GDF-15 in the pathophysiology of early atherosclerosis and MI in the young population.

## 1. Introduction

The leading cause of death in the world is cardiovascular disease (CVD). Acute myocardial infarction (MI) is among the most common causes of death in developing countries [1]. Acute MI in the “young” is a significant problem with a prevalence ranging from 6% to 10% [2]. Most registries and studies use an age range of 40–45 years to define “young” individuals with coronary artery disease (CAD) or acute MI [3]. Traditional differences described in the risk factor profile of younger MI compared to older patients include a higher prevalence of smoking, a family history of premature CAD, and the male sex [4]. Additional non-traditional risk factors, such as substance abuse, thrombophilia, coronary anomalies, immune disease, allergic reactions, and psychological stressors, are also held responsible for the MI of young individuals [3]. While acute coronary syndrome (ACS) rates have dropped among older individuals, comparable reductions have not been seen in the prevalence of younger men experiencing acute MI and cardiovascular events [5].

Over time, researchers have studied various biomarkers and signaling molecules associated with the development of atherosclerosis. An attention-grabbing molecule is growth differentiation factor-15 (GDF-15): a member of the transforming growth factor-beta superfamily [6]. GDF-15 stands as a stress-reactive cytokine that is discharged by diverse cell categories, encompassing endothelial cells, smooth muscle cells, and macrophages. Its function spans over several biological and pathological mechanisms, encompassing inflammation, oxidative stress, and cellular growth [7]. GDF-15 is produced by activated macrophages in response to proinflammatory cytokines like interleukins (IL)—1β, IL-2, tumor necrosis factor-α (TNF-α)—and the macrophage colony-stimulating factor [8]. While GDF-15 typically exhibits a low expression in most tissues under normal physiological conditions, its levels of expression can markedly rise in reaction to pathological stress linked to inflammation or tissue damage [9]. Reactive oxygen species, proinflammatory cytokines, a simulated lack of oxygen, and mechanical stretching can cause an increase in GDF-15 levels in heart muscle cells [10]. Age, smoking, and environmental factors can also increase GDF-15 levels [11]. GDF-15 has a protective role in the regulation of inflammation, endothelial cell function, insulin sensitivity, and weight gain and is cardioprotective in MI [12].

By simple definition, arteria stiffness is the reduced distensibility of the arterial wall, which causes a decrease in the buffering capacity of arteries to the pulsatile cardiac ejection. Arterial stiffness measures, including mainly pulse wave velocity (PWV) and the augmentation index (AIx), represent a significant predictor for upcoming cardiovascular ailment and help in the identification of patients with a higher cardiovascular risk. This holds true regardless of widely recognized cardiovascular risk elements. Elevated arterial stiffness is linked to unfavorable cardiovascular results, regardless of established risk factors like high blood pressure, abnormal lipid levels, diabetes, obesity, advancing age, and tobacco use [13]. Arterial stiffness causes the early arrival of wave reflections in the systole instead of diastole, resulting in an increased systolic afterload and reduced diastolic coronary perfusion pressure. PWV, Aix, and central pressures are related to atheromatic plaque vulnerability, incidence, severity, and the extent of CAD [14]. Kozlov et al. [15] have shown that increased aortic stiffness is a powerful predictor of stable CAD in young and middle-aged men. Arterial stiffness has also been studied in patients with ACS and is suggested to be a strong predictor of major adverse cardiovascular events, including cardiac death, re-acute MI, revascularization, heart failure, and stroke after discharge [16]. In a study conducted in patients with acute MI treated with primary percutaneous coronary intervention, PWV measured within the first week after MI was found to be an independent predictor of infarct size reduction [17]. However, data regarding arterial stiffness in young patients with acute MI are limited.

Increased GDF-15 levels are shown to be related to CVD, such as atherosclerosis and heart failure [18]. The circulating level of GDF-15 increases rapidly in response to cardiovascular injury, such as pressure overload, heart failure, ischemia/reperfusion, and atherosclerosis [19]. Serum GDF-15 levels are significantly increased in CAD patients compared to healthy controls, with a positive correlation shown between GDF-15 and Gensini scores, which suggests that GDF-15 is useful not only in discriminating patients with CAD but also in identifying patients with more severe CAD [20]. Recent studies have also pointed out that GDF-15 is a strong and independent predictor of mortality and recurrent MI in patients with ACS [21]. While GDF-15 is correlated with aortic stiffness and is an effective and important predictor in newly diagnosed hypertension patients [22], there are no current studies exploring the effect of GDF-15 on the development of premature MI.

The aim of this study is to investigate the association between plasma GDF-15 levels and arterial stiffness parameters in patients with premature MI.

## 2. Materials and Methods

### 2.1. Study Population

Forty consecutive patients aged ≤45 years admitted to cardiology clinics with the diagnosis of acute MI were invited to participate in the study. The diagnosis of MI was confirmed via coronary angiography. Patients were evaluated for the presence of CVD risk factors, including hypertension, hyperlipidemia, diabetes mellitus, family history, and cigarette smoking. Hypertension was characterized as having a systolic and/or diastolic blood pressure equal to or exceeding 140/90 mmHg, a prior diagnosis of hypertension, or the utilization of any medications for lowering blood pressure. Diabetes mellitus was defined as having fasting plasma glucose levels exceeding 126 mg/dL for at least three measurements, a previous diagnosis of diabetes, or the use of antidiabetic drugs like oral antidiabetic agents or insulin. Hyperlipidemia was defined as having a total serum cholesterol level of 200 mg/dL or higher, a serum triglyceride level of 150 mg/dL or higher, a low-density lipoprotein (LDL) cholesterol level of 130 mg/dL or higher, a prior diagnosis of hyperlipidemia, or the use of medications to lower lipid levels. Patients with other inflammatory conditions, including systemic lupus erythematosus, typical rheumatoid arthritis, thrombophilia, gout, chronic kidney disease, left ventricular systolic dysfunction, cardiomyopathy, valvular heart disease, and arrhythmias, were excluded from the study. After the exclusion of 10 patients (2 had chronic renal failure, 4 had thrombophilia, 1 had systemic lupus erythematosus, 1 had rheumatoid arthritis, and 2 had bicuspid aortic valve and moderate aortic regurgitation), the remaining 30 patients with premature MI were included in the study. All patients underwent successful revascularization and were discharged with dual antiplatelet therapy, an angiotensin-converting enzyme inhibitor, statins, and a beta blocker. One month after discharge, the patients were called to determine their arterial stiffness parameters and take blood samples for GDF-15 levels.

As a control group, 15 age-/sex-matched volunteers who did not demonstrate symptoms of CAD were included in the study. Volunteers were excluded if they had systemic connective tissue disease, any evidence of systemic infection, arrhythmias, or conduction disorders.

This investigation complied with the principles outlined in the Declaration of Helsinki. This study was approved by the ethics committee of the Marmara University School of Medicine, and all participants gave written informed consent before participating.

### 2.2. Assessment of Arterial Stiffness Parameters

Prior to the study, participants were instructed to abstain from consuming alcohol, coffee, or tea and from eating for a minimum of 12 h. The assessment of arterial stiffness was conducted while participants were in a supine position within a quiet, temperature-controlled room maintained at a temperature range of 22–24 °C during the early morning. Measurements were executed using a Mobil-O-Graph arteriograph system (Mobil-O-Graph NG, Stolberg, Germany).

This system is capable of detecting signals from the brachial artery even when the cuff pressure exceeds the systolic pressure in the brachial artery by 35 mmHg. This methodology relies on the fact that the contraction of the heart muscle generates an initial pulse wave (early systolic peak) that travels through the aorta. This initial wave is reflected from the aortic wall at a specific branching point, giving rise to a second reflected wave (late systolic peak). The shape of this second reflected wave is influenced by the stiffness of the major artery.

By assessing the amplitude and time difference between the first and second waves, parameters such as AIx (adjusted for a heart rate of 75 beats per minute) and PWV were calculated in accordance with current medical guidelines [23]. We performed arterial stiffness measurements once for each study patient.

### 2.3. Assessment of Plasma GDF-15 Levels

Plasma was collected using EDTA-Na2 as an anticoagulant. Samples were centrifuged for 15 min at 1000× *g* at 2–8 °C within 30 min of collection. Supernatants were collected to carry out the assay. Plasma GDF-15 concentration levels were measured using a relevant Human GDF15 ELISA Kit (catalog number: E-EL-H0080, Elabscience Biotechnology Inc., Houston, TX, USA).

### 2.4. Statistical Analysis

All statistical tests were performed via a statistical analysis program (SPSS 21.0 for Windows, Chicago, IL, USA). The distribution of data was tested using a one-sample Shapiro–Wilk test. Categorical variables were defined as a percentage, and comparisons were made using the Chi-square test. Continuous data were expressed as the mean ± standard deviation in the case of a parametric distribution and as a median with 25th and 75th percentiles if they were not distributed normally. Student’s *t*-test was used to compare normally distributed continuous variables, while the Mann–Whitney U test was used to compare the nonparametric continuous variables. Correlation analysis was performed by the Pearson or Spearman test. Logistic regression analysis was performed to determine the independent factors associated with premature MI. A significance level was set at *p* < 0.05.

## 3. Results

Thirty patients (mean age: 39 ± 5 years, 23 male) with premature MI were consecutively included in the study. The characteristics of the study population are shown in Table 1. There were no significant differences in age, sex, or comorbidities between the groups. The laboratory parameters of the groups are shown in Table 2. The patients with premature MI had significantly higher GDF-15 levels compared to the controls. While total cholesterol and LDL cholesterol levels did not show a significant difference between the patients and control group, the patients with premature MI had significantly lower high-density lipoprotein (HDL) cholesterol and significantly higher triglyceride, uric acid and N-terminal pro-B type natriuretic peptide (NT-ProBNP) levels.

The arterial stiffness and hemodynamic parameters of patients and controls are listed in Table 3. There were not any significant differences in PWV and AIx for the patients with premature MI compared to the controls. Patients with premature MI had significantly lower peripheral pulse pressure and heart rates compared to the controls.

While there was not any significant relation between GDF-15 and PWV (r = 0.255, *p* = 0.091), GDF-15 was significantly and positively correlated with triglyceride (r = 0.296, *p* = 0.048) and uric acid levels (r = 0.376, *p* = 0.011) and negatively correlated with HDL (r = −0.368, *p* = 0.013), as shown in Table 4. Logistic regression analysis was performed to determine the independent factors associated with premature MI. In addition to GDF-15, PWV, HDL cholesterol, LDL cholesterol, triglyceride, and uric acid were included in the model. Both GDF-15 (*p* = 0.046, odds ratio: 1.092, 95% confidence interval: 1.003–1.196) and HDL cholesterol (*p* = 0.037, odds ratio: 0.925, 95% confidence interval: 0.859–0.995) were found to be independent factors associated with premature MI.

## 4. Discussion

In our study, we found that GDF-15 was significantly higher in patients with premature MI compared to the controls. To the best of our knowledge, the association between plasma GDF-15 levels and premature MI has not been assessed. On the other hand, PWV was found to be similar between the patients with premature MI and the controls, and we did not find a relation between GDF-15 and PWV measures.

GDF-15 is a stress-responsive cytokine that increases during inflammatory processes. Since inflammatory responses play an important role in atherogenesis and CAD, recent studies have explored the relationship between GDF-15 and CAD. Increased GDF-15 levels are shown to be positively associated with CAD, and GDF-15 has been suggested as a useful adjunct when discriminating against CAD [20]. There are various hypotheses regarding the relationship between the plasma GDF-15 level and CAD. GDF-15 induces endothelial dysfunction by impairing nitric oxide synthesis and promoting endothelial cell apoptosis, which contributes to the initiation and progression of atherosclerotic lesions [24]. In addition, GDF-15 promotes the recruitment and activation of immune cells, particularly macrophages, leading to the formation of foam cells and the development of fatty streaks [7]. Population studies have also demonstrated positive correlations between increased GDF-15 levels and the increased risk of cardiovascular events, emphasizing its potential importance as an independent predictor of mortality in patients with CAD and heart failure [20,25,26,27].

Since GDF-15 is also shown to be correlated to myocardial injury and pressure cardiac overload in animal models, studies have also focused on ACS patients. Similar to the patients with stable CAD, GDF-15 levels are elevated in patients with ACS. In addition, there is also a significant association between high GDF-15 levels and mortality and recurrent MI in patients with ACS [28,29]. While a study suggested the use of GDF-15 in the early risk stratification of patients with acute chest pain [9], another study with a longer follow-up period reported that post-ACS patients who experience a recurrent event have stable and systematically higher GDF-15 levels during 30-days to 1-year follow-up compared to the event-free post-ACS patients [28].

Myocardial infarction represents the final stage within the progression of atherosclerotic pathophysiology. When endothelial function is compromised, factors that promote vasoconstriction, inflammation, proliferation, and thrombosis take precedence, leading to a significantly proatherogenic condition [30]. In our study, we explored the relation between GDF-15 and acute MI in specifically young patients. While a universal definition or standardized set of diagnostic criteria for early-stage MI is lacking, the majority of studies have chosen an age range of 40–45 years to categorize patients as “young” in the context of CAD or acute MI [3,31]. Traditional risk factors for acute MI at a young age include physical inactivity, smoking, alcohol consumption, dyslipidemia, diabetes mellitus, hypertension, and obesity, with smoking as the most dominant [32,33]. Although plaque-based mechanisms dominate the etiology of MI in young individuals [34], non-traditional risk factors, such as drugs and toxins, allergic reactions and hypersensitivity, infections, immune-mediated inflammatory diseases, and thrombophilia constitute another important part of the etiology of premature MI [3]. Due to their close relationship with inflammation and oxidative stress, these traditional risk factors can also cause an increase in GDF-15 levels. A study exploring the difference in GDF-15 levels between young and elderly ACS patients could be useful in the identification of underlying mechanisms in premature MI and its prognostic importance in these patients.

Arterial stiffness serves as a gauge for the elasticity of arteries and the proportional influence of collagen and elastin [35]. Research has indicated that oxidative stress and inflammation are the primary culprits behind the stiffening of blood vessels [36]. Abnormal collagen turnover, cytokines, and metalloproteinase activity are common biochemical links between vascular and myocardial stiffness. GDF-15 is also involved in promoting inflammation and oxidative stress within the arterial wall, exacerbating the atherosclerotic process [24], and can also contribute to the stiffening of the arteries. Sokmen et al. demonstrated that GDF-15 levels are significantly correlated with increased aortic stiffness and have suggested GDF-15 as a significant predictor of aortic stiffness. A study conducted on the Framingham Offspring cohort has also shown a positive correlation between arterial stiffness parameters and GDF-15. However, in a study with elderly hypertensive patients, Barma et al. failed to show the relation between GDF-15 levels and PWV and AIx. Similarly, in our study, we assessed the PWV and AIx of patients to determine the presence of aortic stiffness, and we did not demonstrate a significant association between GDF-15 and PWV or AIx. This could be attributed to different mechanisms underlying acute MI in relatively young patients. Another possible explanation may be the positive effects of medications and lifestyle modifications on arterial stiffness. The patients with premature MI were discharged with optimal medical treatment, including an angiotensin-converting enzyme inhibitor, statins, and a beta blocker. It is well known that certain anti-hypertensive medications, regular exercise, statin use, and smoking cessation can improve arterial stiffness and decrease the PWV value [37]. However, we performed the evaluation of PWV one month after discharge to minimize the possible effects of these medications and lifestyle modifications. Yet, patients with premature MI had a significantly lower heart rate, which is already proof of the efficacy of beta blockers in these patients.

Low HDL cholesterol and elevated triglyceride levels are well-known parameters associated with CAD. A major dyslipidemia can be diagnosed in more than 80% of patients with established premature CAD. These dyslipidemias constitute not only elevations in LDL cholesterol (hypercholesterolemia) but also indicate abnormalities in the metabolism of triglyceride-rich lipoproteins, HDL, and lipoprotein(a). In our study, HDL cholesterol levels were significantly lower, while triglyceride and uric acid levels were significantly higher in patients with premature MI. Li et al. have shown how HDL levels are strongly associated with premature MI, and HDL levels at the onset of MI can predict cardiovascular events in young males [38]. In our study, we demonstrate that, in addition to GDF-15 levels, low HDL cholesterol levels are independent factors associated with premature MI.

Elevated levels of GDF-15 were related to several cardiovascular risk factors, including the male gender, current smoking, body mass index, waist circumference, diabetes, fasting glucose, triglycerides, and low HDL cholesterol. We also found significant correlations between GDF-15 levels and HDL cholesterol, triglyceride, and uric acid levels, which supports the studies that suggest the use of GDF-15 as a clinical biomarker of cardiometabolic risk in the community [39]. Similarly, a recent study conducted to explore the association of the metabolic syndrome and its components with GDF-15 among older adults has shown a close relation between GDF-15 and low HDL cholesterol levels, concluding that the metabolic syndrome is associated with higher GDF-15 levels in older adults with abdominal obesity, hyperglycemia, low HDL cholesterol and inflammation as the main drivers of this association [40]. Uric acid is associated with processes such as higher oxidative stress, inflammation, and endothelial dysfunction [41]. Molecular signals produced using oxidative stress, insulin resistance, endothelial dysfunction, or the inflammatory response allow hyperuricemia to stimulate the onset and progression of CAD and increase the risk of mortality and morbidity from CAD. Cheng et al. have shown a positive correlation between GDF-15 levels and CAD in male patients with hyperuricemia [42]. They found that GDF-15 concentrations were correlated with uric acid, and GDF-15 levels were higher in male hyperuricemia patients suffering from CAD than in those with hyperuricemia alone.

Despite significant advancements achieved in the prognosis of CVD with the management of risk factors like hypertension, diabetes, and dyslipidemia, the burden of CVD remains considerable [43]. It has been widely suggested that these traditional risk factors do not entirely explain the increasing prevalence of CVD, with more than 50% of CVD patients exhibiting none of these risk factors [44]. Furthermore, a substantial number of individuals afflicted by fatal CVD events, including sudden cardiac death, MI, or stroke, do not exhibit preceding symptoms or warning indicators [45]. Hence, the early detection of subclinical atherosclerosis and the identification of individuals with a heightened risk of future CVD is crucial. The number of younger individuals presenting with MI is steeply increasing, and individuals with MI at a young age constitute a special patient population with different clinical features compared to patients with older MI. The underlying mechanisms may be different in premature MI and PWV, which increases with classical cardiovascular risk factors and may not be a suitable method for predicting premature MI. The GDF-15 molecule may have emerged as a valuable tool for assessing and monitoring individuals within this unique cohort of premature MI.

## 5. Study Limitations

The small sample size is the major limitation of our study. Since it was a cross-sectional study and we did not have follow-up data, we could not assess the prognostic importance of GDF-15 in patients with premature MI. We evaluated the arterial stiffness parameters and GDF-15 levels one month after discharge. Although the arterial stiffness of the patients was evaluated one month after discharge, the medications started after MI, including the angiotensin-converting enzyme and statins, and lifestyle modification might have improved the PWV measures of these patients. In addition, the patients might be using additional medication, such as young female patients who have been using hormone therapy or oral contraceptives, which might again influence arterial stiffness parameters. We did not include elderly patients in our study. The addition of a group consisting of elderly patients might help to determine the differences in GDF-15 levels and the possible underlying mechanisms of MI between young and elderly ACS patients. The prevalence of MI is not high in young female patients, and risk factors and underlying mechanisms may be different in premature MI between female and male patients. Due to a small sample size, we could not compare the association of GDF-15 with premature MI between men and women.

## 6. Conclusions

This study provides, to the best of our knowledge, the first evidence that GDP-15 levels are elevated in patients with premature MI. Therefore, it presents a promising prospect as a potentially valuable novel biomarker endowed with the capacity to provide unique prognostic insights and steer the development of effective treatment strategies in these patients. This pilot study may trigger further studies with larger samples and longer follow-up periods to elucidate the central role of GDP-15 in pathophysiology and the prognosis of premature MI.

## Figures and Tables

**Table 1:** Characteristics of the patients and controls.

	Patients (n = 30)	Controls (n = 15)	*p*
Age (years)	39.6 ± 5.3	40.2 ± 3.4	0.694
Gender (male) n (%)	23 (76.7)	11 (73.7)	1
BMI (kg/m^2^)	28.6 ± 5.3	27.2 ± 3.9	0.350
Coronary artery disease (n-%)	7 (21.1)	0	0.77
Hypertension (n-%)	6 (20)	1 (6.7)	0.395
Diabetes mellitus (n-%)	11 (36.6)	3 (20)	0.255
Hyperlipidemia (n-%)	4 (13.3)	0	0.285
Smoker (n-%)	10 (33.3)	6 (40)	0.660
Family history (n-%)	4 (13.3)	2 (13.3)	1
Clinical presentation Anterior STEMI (n-%) Inferior STEMI (n-%) NSTEMI/USAP (n-%)	12 (40) 9 (30) 9 (30)	- - -	

BMI: Body mass index; STEMI: ST-segment elevation myocardial infarction; NSTEMI: Non-ST segment elevation myocardial infarction; USAP: unstable angina pectoris.

**Table 2:** Laboratory parameters of the patients and controls.

	Patients (n = 30)	Controls (n = 15)	*p*
Hemoglobin (g/dL)	14.5 ± 2.1	14.4 ± 2.0	0.838
Hematocrit (%)	41.8 ± 5.0	43.0 ± 5.3	0.470
Creatinine (mg/dL)	0.82 ± 0.15	0.77 ± 0.11	0.323
Total cholesterol (mg/dL) Median (25th and 75th percentile)	170 (153–213)	188 (170–196)	0.159
Triglycerides (mg/dL) Median (25th and 75th percentile)	138 (117–174)	121 (91–132)	**0.020**
LDL cholesterol (mg/dL) Median (25th and 75th percentile)	114 (92–133)	121 (82–131)	0.885
HDL cholesterol (mg/dL) Median (25th and 75th percentile)	37 (29–40)	48 (41–56)	**<0.001**
Uric acid (mg/dL)	5.9 ± 1.4	4.8 ± 1.0	**0.007**
NT-ProBNP (ng/mL) Median (25th and 75th percentile)	0.22 (0.20–0.25)	0.18 (0.17–0.23)	**0.004**
GDF-15 (Pg/mL) Median (25th and 75th percentile)	38.1 (24.7–72.6)	21.4 (21.0–27.6)	**0.002**

LDL: low-density lipoprotein; HDL: high-density lipoprotein; NT-ProBNP: N-terminal pro-B-type natriuretic peptide; GDF-15: Growth Differentiation Factor-15. Data were expressed as the mean ± standard deviation when they were distributed normally and compared with Student *t* test. Data were expressed as the median with the 25th and 75th percentiles if they were not distributed normally and compared with the Mann–Whitney U test.

**Table 3:** Comparison of arterial stiffness parameters and hemodynamic parameters among groups.

	Patients (n = 30)	Controls (n = 15)	*p*
Peripheral systolic blood pressure (mmHg)	115 ± 16	123 ± 11	0.115
Peripheral diastolic blood pressure (mmHg)	77 ± 13	78 ± 11	0.656
Peripheral mean blood pressure (mmHg)	95 ± 11	98 ± 11	0.455
Peripheral pulse pressure (mmHg)	38 ± 9	44 ± 9	**0.037**
Heart rate (beat/min)	75 ± 12	83 ± 10	**0.023**
Cardiac output (L/min)	5.0 ± 0.6	4.9 ± 0.8	0.419
Cardiac index (L/min/m^2^)	2.6 ± 0.4	2.6 ± 0.5	0.762
Central systolic blood pressure (mmHg)	107 ± 15	113 ± 11	0.094
Central diastolic blood pressure (mmHg)	78 ± 13	80 ± 11	0.515
Central pulse pressure (mmHg)	29 ± 8	33 ± 7	0.088
Reflecting magnitude (%)	61 ± 11	61 ± 6	0.819
Augmentation index (%)	14 ± 10	18 ± 8	0.117
PWV (m/s)	6.04 ± 0.8	6.09 ± 0.7	0.700

PWV: Pulse wave velocity.

**Table 4:** Correlation of GDF-15 levels with PWV, uric acid, HDL and triglyceride levels.

GDF-15	PWV	Uric Acid	HDL	Triglyceride
*p*	0.091	**0.011**	**0.013**	**0.048**
r	0.255	0.376	−0.368	0.296

GDF-15: Growth differentiation factor-15; PWV: pulse wave velocity; HDL: high-density lipoprotein.

## Data Availability

Certain confidentiality constraints prevent us from disclosing the underlying database used in this study.
